# Androgen Effects on Neural Plasticity

**DOI:** 10.1089/andro.2021.0022

**Published:** 2021-12-23

**Authors:** Nariko Kuwahara, Kate Nicholson, Lauren Isaacs, Neil J. MacLusky

**Affiliations:** Department of Biomedical Sciences, University of Guelph, Guelph, Ontario N1G 2W1, Canada.

**Keywords:** testosterone, neurosteroidogenesis, hippocampus, synaptogenesis, glia

## Abstract

Androgens are synthesized in the brain, gonads, and adrenal glands, in both sexes, exerting physiologically important effects on the structure and function of the central nervous system. These effects may contribute to the incidence and progression of neurological disorders such as autism spectrum disorder, schizophrenia, and Alzheimer's disease, which occur at different rates in males and females. This review briefly summarizes the current state of knowledge with respect to the neuroplastic effects of androgens, with particular emphasis on the hippocampus, which has been the focus of much of the research in this field.

## Introduction

The incidence and progression of several neurological and psychiatric disorders, including Alzheimer's disease (AD),^1^ schizophrenia,^2^ and depression,^3^ are sexually differentiated. For example, in comparison to their male counterparts, women are more likely to develop AD and experience a more rapid decline in memory.^4^ The severity of cognitive symptoms associated with many of these disorders also appears to differ between the sexes.^2^ That these sex differences might be attributable, at least in part, to the actions of sex steroid hormones has prompted increased interest in the mechanisms mediating hormonal effects on the brain.

Much of the work in this field has focused on the effects of the principal ovarian steroid, estradiol, because of data indicating that circulating and locally synthesized estradiol may play a fundamental homeostatic role in the brain.^5–7^ In addition to wide-ranging effects on neurotransmitter and neuropeptide systems^8,9^ and cerebral energy metabolism,^5^ estradiol has also been demonstrated to induce dramatic, reversible changes in cellular morphology in many regions of the brain. Estrogen-induced changes in neuroplasticity include alterations in dendritic morphology, spine synapse density, and neuron number.^10,11^

Relatively less is known about the effects of androgens on the brain, although there are similarities between their effects and those of estrogens, particularly in females, at least in part because testosterone is a substrate for estradiol biosynthesis in the brain.^12^ Androgen-induced changes in cognitive functions and behavior have been reported in humans^13–15^ as well as experimental animals.^16,17^ In men, the gradual decline in testosterone with age has been correlated with cognitive performance, with lower levels of free testosterone in older men being associated with reduced performance on verbal and spatial memory tasks.^18–20^

In male rodents, gonadectomy and testosterone supplementation has been found to influence spatial and working memory, although the responses appear to be mixed, possibly due to methodological differences across studies, including the type of cognitive test used and age of the animals.^16^ Associated with these effects, morphological analysis has demonstrated that androgens induce marked changes in hippocampal and cerebral cortical dendritic morphology, spine and spine synapse density, and hippocampal neurogenesis.^21–26^

Although the catalogue of reported neuroplastic effects of androgens on the brain continues to expand, how these effects are mediated remains only partially understood. In this review, we provide a brief summary of the current research on androgens and their influence on various aspects of neuroplasticity, with a particular focus on the effects of these hormones in the hippocampus and the possible mechanisms mediating these effects. We also highlight areas of uncertainty and suggest possible directions for future research.

## Androgens in the Brain: An Overview

**FIG. 1.**
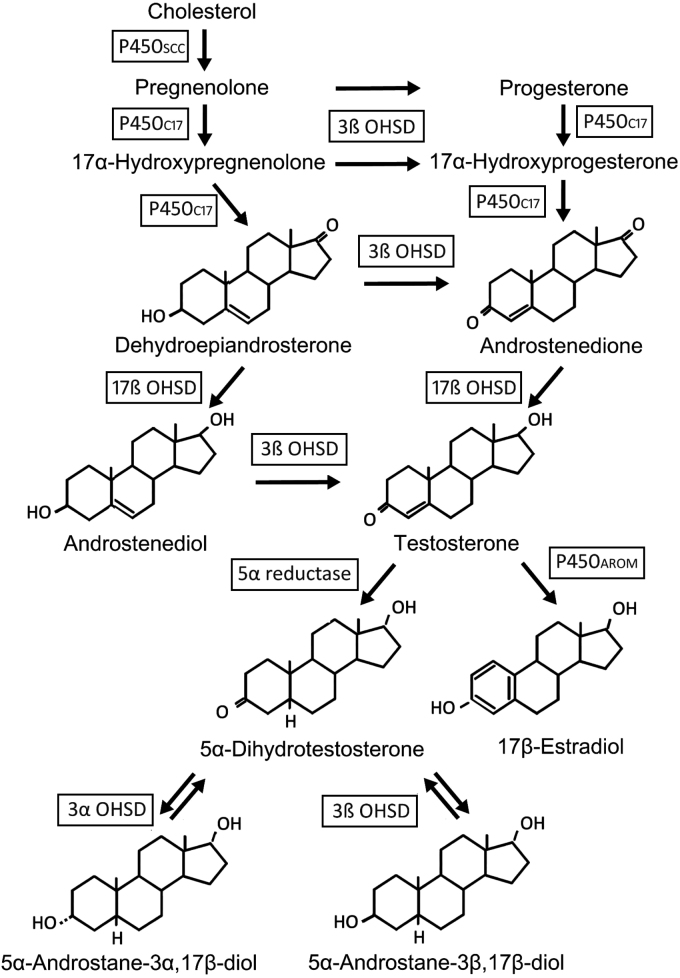
Schematic illustration of the metabolic pathways involved in the biosynthesis and metabolism of the principal physiological androgens. Androgens and their major biologically active metabolites are shown with their structures; steroidal precursors for androgens are shown only by name. The enzymes mediating each metabolic conversion step are indicated by abbreviations in rectangular boxes. 17ß OHSD, 17ß hydroxysteroid dehydrogenase; 3ß OHSD, 3ß hydroxysteroid dehydrogenase; 3α OHSD, 3α-hydroxysteroid dehydrogenase; 5α reductase (3-oxo-5α-steroid 4-dehydrogenases, a family of isozymes with different tissue distributions^158^); P450_AROM_, aromatase, or CYP19; P450_C17_, 17α-hydroxylase/17,20 lyase or CYP17; P450_SCC_, cholesterol side chain cleavage enzyme, or CYP11A1.

Androgens, similar to other steroid hormones, are derived from the metabolism of cholesterol (Fig. 1). The initial, rate-determining step in steroidogenesis is cleavage of the cholesterol side chain by the P450scc enzyme, associated with the inner mitochondrial membrane, to form pregnenolone. Pregnenolone acts as a substrate for the synthesis of other steroid hormones via conversion to either 17α-hydroxypregnenolone or progesterone.

These steroids are precursors for the synthesis of dehydroepiandrosterone (DHEA), androstenediol, and androstenedione, weak androgens that are themselves substrates for conversion to testosterone, the predominant androgen circulating in the bloodstream. In androgen target tissues, testosterone is further metabolized to a range of biologically active metabolites, including 5α dihydrotestosterone (DHT), via the actions of 5α-reductase, as well as estradiol via cytochrome P450-aromatase.

In addition to being a more potent androgen than testosterone, DHT is also the precursor for two other biologically active metabolites: 5α-androstane-3α,17β-diol (3α-diol) and 5α-androstane-3β,17β-diol (3β-diol). 3α-diol is an allosteric modulator of the GABA_A_ receptor.^27^ Both 3β-diol and, to a lesser extent 3α-diol, are ligands for estrogen receptor β (ERβ), possibly mediating some of the analgesic, anxiolytic, and cognitive-enhancing effects of circulating testosterone.^28^

This pattern of local tissue metabolism complicates elucidation of the mechanisms involved in mediating the effects of testosterone on the brain, since not only may multiple different neuronal and glial cell types be involved, but also there may be contributions from multiple receptor systems, responding to locally produced metabolites with a range of bioactivities. More detail regarding the enzymes and pathways involved in steroidogenesis is provided elsewhere.^29,30^

Although testosterone is often considered to be primarily a male sex hormone, it is present in females as well as males and exerts important physiological effects in both sexes.^31^ The majority of circulating testosterone is synthesized by the Leydig cells of the testes in males and in the ovarian stroma in females,^32^ although the amounts produced by the testes are ∼7 to 8 times higher than those produced by the ovaries.^31^ Biosynthesis of testosterone also occurs in the cortex of the adrenal gland in both sexes.^33,34^

Since androgens are nonpolar and are capable of easily diffusing across the blood–brain barrier, it was long assumed that androgens were synthesized in the periphery and passively distributed throughout the brain to exert their effects.^35^ However, extensive evidence indicates that the brain itself is capable of local steroid synthesis.^6,12,36^ This can occur by either local metabolism of circulating steroid intermediates or via *de novo* synthesis from cholesterol (reviewed in Schmidt et al.^37^).

The idea that androgens could be synthesized in the brain was first developed by Baulieu and colleagues in the 1980s. These authors found that androgens (principally the adrenal androgen DHEA) and their sulfoconjugate were present in higher concentrations in the nervous system than in the plasma, and these high levels remained long after gonadectomy and adrenalectomy of male rats.^38^

These findings led to the introduction of the term “neurosteroids” to refer to steroids synthesized locally in the brain.^36,38^ Subsequently, the presence of the necessary steroidogenic enzymes in the brain was established in several species, including rodents and humans (reviewed in^39–42^). Along with the enzymes required for conversion of the primary sex steroids into biologically active metabolites.^36,43–45^ the discovery of *de novo* neurosteroid synthesis has greatly expanded our appreciation of the diverse roles of gonadal steroids in the regulation of neuronal structure, function, and activity.^46^

Androgen action on the brain involves effects mediated via both steroid receptors of the nuclear receptor family, which bind to hormone response elements on DNA to regulate the expression of target genes,^47,48^ and more rapid effects initiated via membrane receptors linked to kinase signaling cascades.^49^ Nuclear steroid receptors (including the “classical” estrogen and androgen receptors [AR]) may in some instances subserve both roles, with a portion of the receptors in the cell being translocated to the plasma membrane.^50^

The AR have been found in both nuclear and extranuclear sites in hippocampal neurons,^51,52^ as well as on oligodendrocytes and astroglia.^53^ Estradiol, whether synthesized locally or delivered via the circulation, acts on both nuclear estrogen receptors (ERα and ERβ) and the G-protein coupled membrane estrogen receptor 1,^54^ which are present in several subregions of the hippocampus and dentate gyrus (DG).^55,56^

## Androgens and Long-Term Potentiation

Electrophysiological studies of long-term potentiation (LTP) in hippocampal slice preparations provided the first indication that androgens might induce functional plasticity in the hippocampus. LTP and long-term depression (LTD) are lasting changes in synaptic response following an initial stimulus that are believed to reflect key molecular mechanisms underlying learning and memory.^57–59^ Gonadal hormones such as androgens have a profound influence on the induction of LTP and LTD in the hippocampus.^60^

After orchidectomy, male mice and rats displayed a reduction in LTP and a decrease in the amplitude of field excitatory postsynaptic potentials (fEPSP).^58,61^ Orchidectomy also altered paired-pulse facilitation of fEPSP within the hippocampus,^61^ suggesting that the removal of circulating androgens has a significant effect on presynaptic transmission, as well as on synaptic maturity.^61^ These effects are multifaceted, including both positive and negative effects on hippocampal neural activity.

Thus, Skucas et al.^62^ found that in adult male rats, orchidectomy resulted in increased synaptic transmission and LTP in the mossy fiber pathway, associated with a long-lasting upregulation of mossy fibers brain-derived neurotrophic factor (BDNF) immunoreactivity, compared with sham-operated rats.^62^ Thus, the overall effects of androgen on hippocampal function may be mixed, enhancing some aspects of hippocampal neurotransmission while simultaneously limiting hyperexcitability and some forms of plasticity in the mossy fiber–CA3 pathway.^62^

In addition to orchidectomy-based studies, pharmacological and molecular targeting of the AR has also been used to investigate the effects of androgens on hippocampal LTP and LTD. Although administration of the AR antagonist flutamide has minimal effects on hippocampal LTP, it impairs the induction of LTD of CA1 pyramidal neurons, indicating a disruption in signaling processes.^60,63^ The AR deletion in an AR mutant mouse model demonstrated a reduction in N-Methyl-D-aspartate (NMDA) receptor-mediated EPSP and high-frequency stimulation LTP of CA1 pyramidal neurons, indicating impairments in glutamate receptor activation.^64^

Although NMDA receptor levels did not differ between AR mutant and wild-type mice, this suggests that eliminating AR action leads to an overall decrease in the activation and function of NMDA receptor.^64^

The administration of androgens such as testosterone and its major 5α-reduced metabolites DHT and 3α-diol has also been shown to directly affect hippocampal synaptic transmission. *Ex vivo* administration of testosterone has been reported to facilitate LTP, increase overall excitability of CA1 pyramidal neurons in both gonadectomized male and female rats, and normalize CA3 mossy fiber fEPSP of orchiectomized males back to levels comparable to those observed in intact rats.^58,61,62^ DHT has effects on LTP similar to those of testosterone, suggesting that local conversion to estradiol is not required for the response.

Treatment with the 5α-reductase inhibitor finasteride to block the metabolism of testosterone to DHT and 3α-diol in gonadally intact males resulted in a reduction in LTD and decreased fEPSP of CA1 pyramidal neurons.^65^ This response was reversed with the application of DHT, but not testosterone.^65^ DHT has been shown to reverse increases in mossy fiber transmission of orchiectomized rats back to the levels observed in sham-operated animals.^62^ However, 3α-diol had no effect on CA3 fEPSP,^62^ consistent with the view that the effects were mediated via AR-dependent mechanisms.

## Androgens and Neurogenesis

The electrophysiological evidence for functional alterations in hippocampal neuroplasticity after androgen exposure led to studies aimed at defining the effects of these hormones on the hippocampal circuitry. Studies over the past 40 years have demonstrated that neurogenesis continues in regions of the adult vertebrate brain that are involved in learning and memory, notably in the vocal control centers of songbirds^66^ and the DG of the hippocampus.^67^ Newly proliferated neurons in the DG subgranular zone migrate to the inner granule cell layer, where they extend long axons along the mossy fiber pathway to the CA3 subregion.^68–70^

Neurogenesis is a complex multistep process that involves the proliferation, differentiation, migration, and survival of new neurons.^71,72^ A growing body of research indicates that, in the hippocampus of adult rodents, gonadal steroids affect multiple steps in this pathway. This subject is covered in detail in the article by Blankers and Galea in this special issue,^21^ so we will only provide a brief summary here.

The effects of gonadal steroids on neurogenesis appear to be both sex- and hormone-specific. Thus, estradiol modulates new neuron survival and cell proliferation in the DG of adult female, but not male, rats,^73^ whereas the reverse is true for the non-aromatizable androgen, DHT.^74^ By contrast, in adult male voles, testosterone appears to enhance the survival of newly generated neurons in the DG, with no significant difference in cell proliferation.^75^

Similarly, castration of adult male rats led to a decrease in cell survival of new neurons within the DG, with no effect on cell proliferation.^24^ However, in castrate adult male rats, 30 days of hormone replacement via injections or implants of testosterone propionate have been shown to increase neurogenesis in the DG via cell survival, relative to controls.^24,76^ The dose and duration of testosterone administration appears to be an important determinant of the effects of testosterone on neurogenesis.^24,77^

Studies using metabolites of testosterone, as well as receptor-specific antagonists, suggest that the effects of testosterone on neurogenesis in the male hippocampus are probably mediated via AR, as opposed to local estrogen biosynthesis.^24,76^ Consistent with this hypothesis, Okamoto et al. found that in male rats, mild exercise increased local intrahippocampal DHT synthesis, resulting in significantly increased neurogenesis, a response blocked by systemic administration of the AR antagonist, flutamide.^78^

The molecular mechanisms underlying these effects are not yet fully understood. Some studies in male rodents have detected AR protein and mRNA expression in the DG,^51,79^ suggesting that androgens may influence cell survival directly via AR in newly generated neurons. However, whether the effects are actually mediated directly within the DG remains uncertain because of evidence indicating that AR are expressed in the CA1 and CA3 subregions of the hippocampus, but not the DG.^80–83^

Because AR are not expressed by immature neurons within the DG of young male mice and rats, it is possible that their effects may be mediated indirectly via target cells located elsewhere in the brain.^76,84^ In particular, androgens may bind to AR on CA3 pyramidal cells, initiating the release and retrograde transport of a survival factor that may act on newborn neurons in the DG.^74,76,85^ This hypothesis is consistent with observations indicating that newly formed neurons in the DG send projections to CA3, whereas damage to the CA3 has significant effects on neurogenesis in the DG.^86,87^

It also provides a striking parallel to work in the developing spinal cord, in which motoneurons in the spinal nucleus of the bulbocavernosus (SNB) are dependent for survival on androgen-dependent trophic support mechanisms via the target muscles that they innervate, even though the SNB neurons themselves do not express AR.^88^

## Androgens and Hippocampal Synaptic Plasticity

A large body of evidence has demonstrated the effects of androgens on hippocampal plasticity, which may play important roles in normal physiological responses to changes in the environment. For example, in response to a stressful stimulus, males exhibit higher hippocampal CA1 dendritic spine density in comparison to their female counterparts.^89^ These rapid synaptic modulations may be mediated by sex steroids such as androgens because of their local and rapid synthesis within the brain.^90,91^ Local synthesis results in elevated DHT, testosterone, and estradiol levels within the hippocampus, when compared with plasma levels.^90,92^

The majority of studies, however, have not been based on examination of the effects of physiological variations in steroid levels, but rather on experiments in which gonadal hormone levels are controlled by surgical gonadectomy and hormone replacement. Morphological studies often correlate changes in dendritic spine density with spine structure and the numbers and structure of spine synaptic contacts.^93^ After orchidectomy, studies in both rats and non-human primate St. Kitts vervet monkeys demonstrated a significant reduction in dendritic spine synapse density in the CA1 region of the hippocampus.^22,23^

**FIG. 2.**
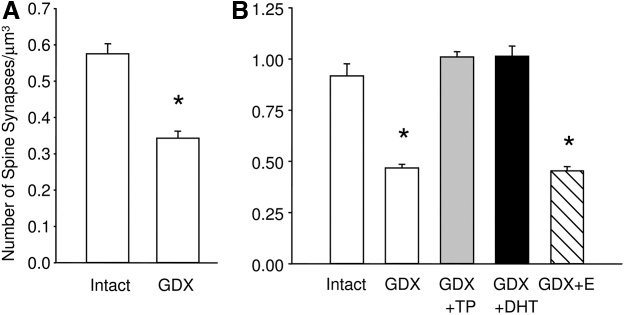
**(A)** Bar graph shows the significant reduction in the number of spine synapses in the CA1 subfield of the hippocampus in GDX monkeys compared with intact monkeys. **(B)** Bar graph shows spine synapse densities in the stratum radiatum of the CA1 region in intact, GDX, GDX and testosterone propionate-treated (GDX+T), GDX and DHT-treated (GDX+DHT), or GDX and estradiol-treated (GDX+E) male rats. Spine synapse density was significantly lower in both GDX and GDX + E animals compared with controls, whereas there was no significant difference between intact animals and GDX animals treated with testosterone-propionate or DHT. Taken from MacLusky et al.^94^. DHT, dihydrotestosterone; GDX, gonadectomized.

In the St. Kitts vervet monkeys, as much as 40% of spine synapse volumetric density was lost after orchidectomy.^23^ Subsequently, it was demonstrated that androgen replacement restores the loss of spine synapse density in the male hippocampus that occurs after surgical gonadectomy^22,94^ (Fig. 2). A similar effect has been reported in the CA1 subfield of the ovariectomized female rat hippocampus treated with testosterone.^23^ However, despite the qualitatively similar nature of the CA1 synaptic response to testosterone in males and females, the underlying mechanisms are probably different.

When orchidectomized rats were replaced with testosterone, DHT or estradiol, only testosterone and DHT reversed the orchidectomy-induced loss of synapse density, whereas estradiol had no significant effect.^22^ By contrast, in females DHT induced responses similar to those of estradiol, whereas the effects of both testosterone and DHEA on hippocampal synapse density were reversed by concomitant treatment with the aromatase inhibitor, letrozole.^23^ This suggests that in males, both testosterone and DHT can stimulate spine synapse formation independently of aromatization, whereas in females the synaptic response to circulating testosterone is largely mediated via local estrogen biosynthesis.^22,94^

**FIG. 3.**
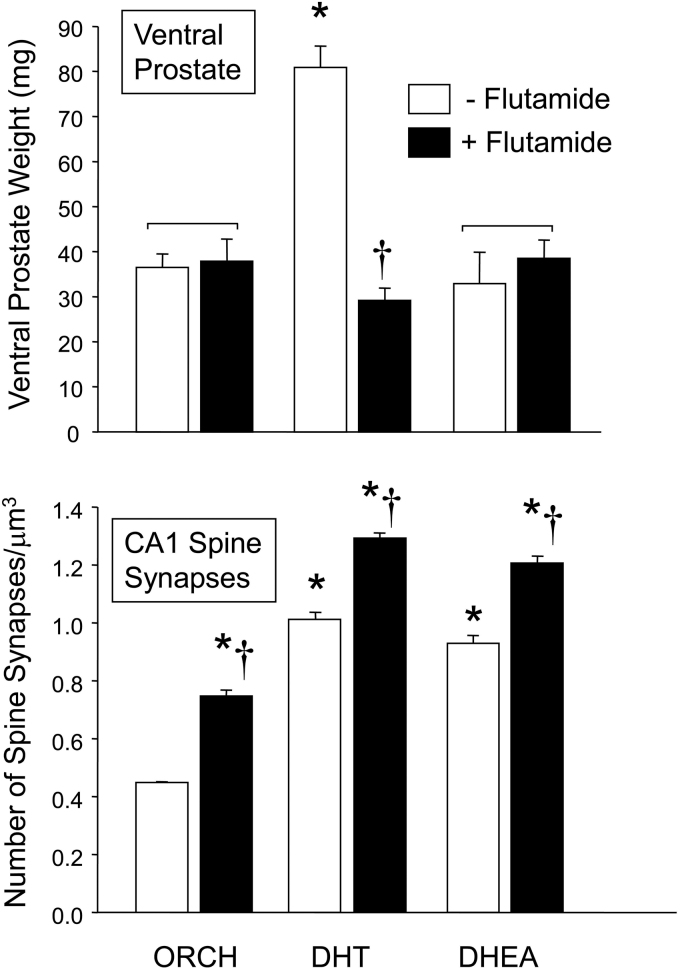
Effects of DHT, DHEA, and flutamide on ventral prostate weight (top panel) and CA1 hippocampal spine synapse density (lower panel) in GDX male rats. *Significantly different from orchidectomized males given the same dose of androgen or oil vehicle; ^†^Significantly different from orchidectomized males treated with the same doses of flutamide or vehicle, without androgen (Scheffe's test; *p* < 0.05 level). Compiled from data in MacLusky et al.^94,95^. DHEA, dehydroepiandrosterone.

These findings cannot, however, be interpreted as indicating that the effects of testosterone on hippocampal synaptogenesis are mediated entirely via the cell nuclear AR system. Studies using the AR antagonist flutamide and animals expressing mutated forms of the AR have raised the possibility that other mechanisms may be involved. Thus, the administration of flutamide to orchidectomized male rats significantly increased CA1 spine synapse density compared with vehicle-treated controls, even though no significant trophic effect was observed in the ventral prostate gland, a widely used marker of androgenic biological activity (Fig. 3).

Flutamide did not block the synaptoplastic effects of either DHT or DHEA: Indeed, the responses to flutamide and the two androgens appeared to be additive, rather than inhibitory.^94,95^ Further emphasizing the complex nature of this response, in testicular feminization mutant (Tfm) male rats expressing defective AR, results with both androgen and antiandrogen treatment were indistinguishable from those observed in wild-type male controls.^96^ This suggests that at least in the CA1 region of the hippocampus, the synaptic effects of testosterone are retained even under conditions in which nuclear AR function is impaired.

This cannot be ascribed to local conversion of the androgen to estradiol, since estradiol does not affect hippocampal synapse density in males^22^ whereas flutamide also exerts additive effects on CA1 synaptogenesis in combination with the non-aromatizable androgen, DHT.^95^

A definitive explanation for these observations remains lacking. One possibility is that the effects of flutamide may involve mechanisms other than those activated by the cell nuclear AR. As previously noted,^95^ flutamide is not a completely specific antagonist of AR-mediated responses and may itself exert some agonist effects. Under *in vitro* cell culture conditions, the main bioactive metabolite of flutamide, hydroxyflutamide, has been reported to activate the mitogen activated protein kinase (MAPK)/extracellular regulated protein kinase (ERK) pathway, even in AR-negative cell lines.^97^

It also exerts weak benzodiazepine-like effects in the mouse brain.^98^ Membrane-associated AR may be involved. Sato et al.^99^ have reported that the self-reinforcing behavioral effects of androgen appear to involve membrane AR. These effects are preserved in Tfm rats, suggesting that membrane receptor-mediated effects of androgens are retained in these animals despite the deficit in nuclear AR-activated responses.^99^ Similar considerations may apply to androgen-induced synaptogenesis, which could explain the continued expression of normal androgen-induced increases in hippocampal synaptogenesis in Tfm males.

## Cellular Mechanisms: How are the Neurotrophic Effects of Androgen Mediated?

The downstream signaling pathways mediating the neuroplastic effects of testosterone and its metabolites also remain only partially resolved. There are two main possibilities: first, that androgens may act directly on neurons to regulate intracellular neuronal signaling pathways contributing to growth and differentiation; and second, that androgen action on other cells, including both neurons and glia, may affect neuronal structure and function indirectly, via neurotrophic and metabolic response mechanisms. These two possibilities are, of course, not mutually exclusive and both may well contribute to testosterone action *in vivo*.

That androgens can act directly on neurons to modulate neuroplasticity has been established by a number of *in vitro* studies, using neurons in tissue culture or hippocampal tissue slices. Androgen treatment increases spine formation in hippocampal neurons in culture.^93,100,101^ Using hippocampal slices from male adult rats, 2-hour treatment with DHT resulted in a significant increase in the densities of middle and large-head spine whereas 2-hour treatment with testosterone resulted in a significant increase in the density of small head spines.^93^

Incubation with letrozole did not affect testosterone's induction of spine density, suggesting that these effects may be attributed to its androgenic effects, as opposed to local conversion to estradiol.^93^ Using primary cultures of rat hippocampal neurons, Guo et al. demonstrated that *in vitro* testosterone and bovine serum albumin-conjugated testosterone treatment similarly resulted in a rapid increase in spine density, consistent with the hypothesis that these effects may be mediated via membrane-associated AR.^101^

**FIG. 4.**
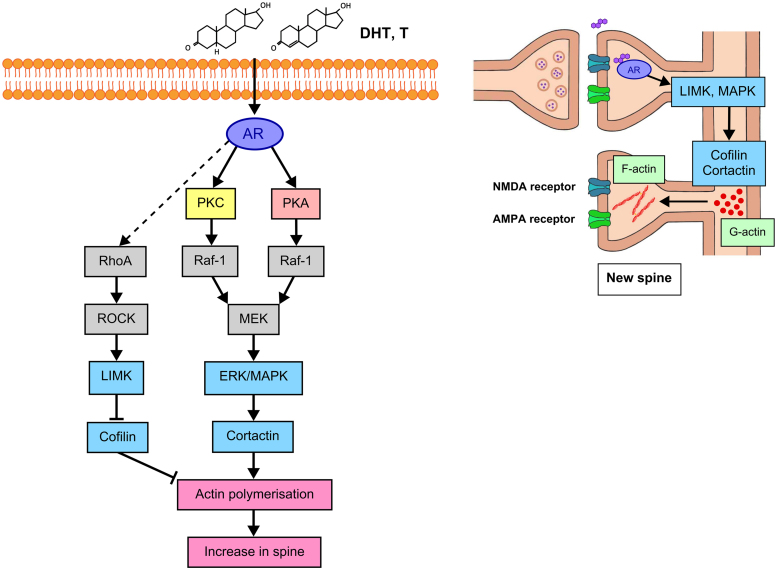
Schematic illustration of androgen-induced spine formation mediated by AR and its downstream kinase networks (adapted from Fig. 5 in Hatanaka et al.^93^). AR, androgen receptors.

In the CA1 subregion of the hippocampus, a number of serine/threonine kinase signaling pathways have been implicated in mediating rapid structural responses to gonadal steroids. Intracellular signaling cascades that have been shown to be differentially activated by sex steroids include the MAPK/ERK, the cyclic AMP/protein kinase A (cAMP/PKA) pathways and the phosphoinositide 3-kinase/PI3K/Akt/mammalian target of rapamycin (PI3/Akt/mTOR) pathway.^54,102–105^ By modulating intracellular signaling cascades, sex steroids may continuously regulate neuronal structure, dendritic spine density, and the functional plasticity of the hippocampal circuitry^62,93,106^ (Fig. 4).

MAPK cascades are well known for their roles in synaptic modulation, and activation of PKC has been shown to be necessary for LTP in hippocampal slices.^107–110^ Androgen-mediated increases in spine density have been correlated with increased expression of structural proteins such as synaptophysin and activation of ERK and cAMP-response element binding protein (CREB).^101^

For many years, it has been known that androgens stimulate rapid activation of PKC and ERK MAPK phosphorylation in the hippocampus and other tissues.^111–114^ In cultured hippocampal neurons, PKC activation has been shown to rapidly upregulate NMDA receptor-mediated signaling^115^ whereas multiple PKC isoforms are expressed within dendritic spines themselves.^116^ Activation of PKC has been shown to induce activation of extracellular receptor kinase and rapid formation of new dendritic spines in cultured hippocampal neurons.^109^

ERK-1 and ERK-2 have been shown to be transiently phosphorylated after AR activation, and this has been implicated in mediating the neuroprotective effects of androgens such as testosterone and DHT.^111^ New spine formation has been associated with increased functional synaptic connectivity and network activity in cultured neurons.^109^ Within CA1 specifically, it has been shown that ERK MAPK mediates synaptic modulation of LTP through activation of both PKA and PKC mechanisms.^110^ Although AR-dependent PKC and ERK activation has been well established in a variety of tissues,^112–114^ findings from Hatanaka et al.^93^ suggest that AR activation by DHT and testosterone results in activation of both PKA- and PKC-mediated ERK signaling.

Within dendritic spines, concurrent expression of these kinase cascades and ARs suggests that they may associate post-synaptically.^51^ Androgen-mediated activation of ERK signaling is further supported by the data of Guo et al.,^101^ who showed that in the presence of U0126 (a selective inhibitor of MAPK/ERK kinases 1 and 2), rapid testosterone-mediated increases in spine density were reversed and subsequent CREB phosphorylation was inhibited, suggesting that this rapid effect is mediated through AR-dependent ERK signaling.^101^ Similarly, testosterone and DHT-induced dendritic spine formation is reduced by inhibition of ERK MAPK, PKA, PKC, or LIMK, whereas inhibition of CAMKII or PI3K has been shown to have no effect on total spine density.^93^

The CREB is an important downstream transcriptional effector of MAPK/ERK activation.^117,118^ CREB activity has been shown to be regulated by many other cell signaling mechanisms, including PI3K/Akt,^119^ PKA,^120,121^ CaMKIV,^122,123^ and PKC.^110^ Coupling of the PKA and PKC to CREB phosphorylation is mediated by the MAPK cascade within hippocampal CA1.^110^

Although CREB has been shown to be rapidly activated by DHT in hippocampal neurons, Nguyen et al. investigated the upstream signaling mechanisms responsible for this and determined that DHT mediates CREB phosphorylation through AR-dependent activation of PKC.^124^ Intriguingly, subsequent studies demonstrated that androgen-induced formation of dendritic thorns within the CA3 stratum lucidum occurs via MAPK and PKC but independently of PKA signaling.^100^

### Indirect mechanisms

Under *in vitro* conditions, tissue culture studies suggest that AR-mediated neuroplastic responses can be initiated directly in neurons; however, under *in vivo* conditions, lesion studies have demonstrated that androgen effects on neurogenesis and synaptic plasticity are at least partially dependent on afferent input from other areas of the brain.^86,87,125,126^ Indirect androgen mediated modulation of cellular responses could theoretically involve synaptic/neurotransmitter input, and/or trophic factors released into the extracellular milieu.

A likely mediator of at least some androgen effects is the neurotrophin BDNF. BDNF and its downstream targets such as post-synaptic density protein 95 (PSD-95) play important roles in regulating spine formation. BDNF modulates functional synaptic properties in the mature and developing nervous system,^127,128^ as well as hippocampal LTP.^129^ Several studies have investigated androgen regulation of BDNF in the context of AR activation and synaptogenesis. For example, Jia et al. determined that testosterone treatment significant increased CA1 spine density, BDNF, PSD-95, and CREB levels, whereas these endpoints were reduced in animals treated with flutamide.^130^

Conversely, orchidectomy has been reported to reduce protein levels of PSD-95 and BDNF in the CA1 region of the hippocampus in male mice, an effect reversed by testosterone replacement.^131^ In other regions of the hippocampus, interactions between androgens and BDNF may have different consequences. Thus, after orchidectomy in rats, Skucas et al. reported increased synaptic transmission and LTP of the mossy fiber pathway along with increased BDNF levels, suggesting that androgens may play an inhibitory role in CA3 to prevent hyperexcitability and aberrant mossy fiber synaptic transmission.^62,132^

Within the cell, BDNF appears to be involved in regulating the distribution of key synaptic proteins that mediate actin cytoskeletal development.^133^ For example, cortactin redistribution has been shown to be the result of a balance between BDNF and NMDA receptor activation during the development of the postsynaptic actin cytoskeleton.^133^ Cortactin is an important structural protein that is known to be a target of ERK activation.

Once phosphorylated, cortactin may play an important role in spine reorganization and remodeling by grouping actin cytoskeletal matrices.^133^ Cortactin contains multiple phosphorylation sites that may be phosphorylated by kinases such as PKA or PKC, which have been shown to be activated after DHT or testosterone treatment.^134^ However, multiple cortactin phosphorylation sites may need to be activated to mediate androgen-induced spine formation.^93^

### Potential contributions from glial-neuronal interactions

Astroglia are critical regulators of synaptic plasticity and neurotransmission.^135^ The potential importance of these interactions is illustrated by data indicating that defective neuronal–glial interactions may play a key role in the etiology of common neurological disorders such as depression,^136^ schizophrenia,^137^ and AD.^138^

Not only are astroglia capable of androgen and estrogen biosynthesis,^139^ but they may also contribute to androgen-mediated regulation of neural plasticity.^140^ Androgen and estrogen receptors are expressed in astrocytes,^141–143^ whereas oligodendrocytes express ERβ, which is involved in estrogen-mediated enhancement of myelination.^144^ In rats, at least after intrahippocampal injury, microglia also express immunoreactive AR.^142^

**FIG. 5.**
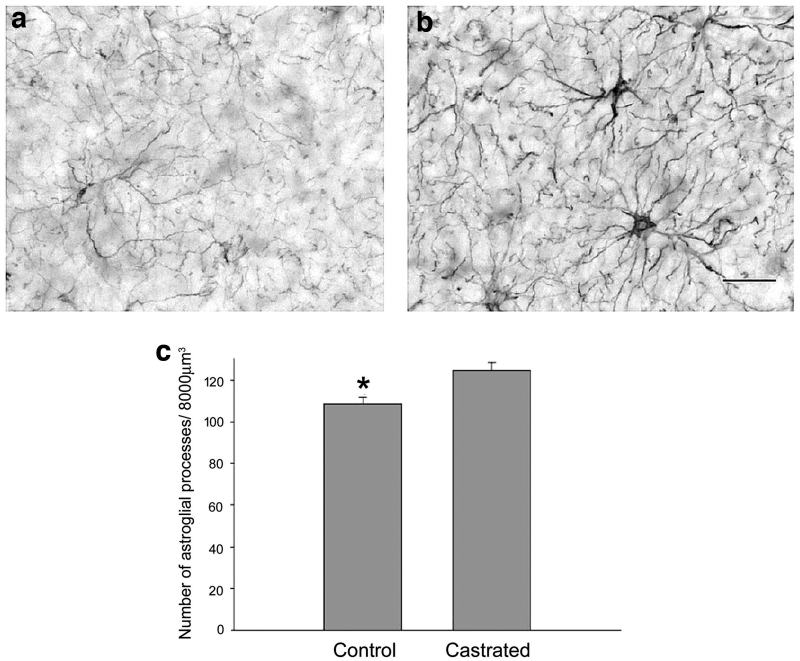
Light micrographs show GFAP-immunoreactive glia and processes in vibratome sections taken from the stratum radiatum of the CA1 hippocampal subfield of a control **(a)** and a 1 month orchidectomized monkey **(b)**. Note the higher density of immunoreactive glia profiles in the hippocampus of the GDX animal **(b)**. Scale bar = 50 μm. **(C)** Bar graphs show the result of a semiquantitative calculation on the density of glia processes in the stratum radiatum of the CA1 hippocampal subfield. The surface density of glia processes is significantly higher (15%) in the orchidectomized (1 month) than control monkeys (mean ± SEM, *p < 0.031, *t* = −3.270, d.f. = 4). GFAP, glial fibrillary acidic protein.

Although the study of androgen modulation of glial–neuronal interactions remains relatively limited, a few studies have reported the effects of testosterone on astroglial morphology. In male rats, Leranth et al.^23^ demonstrated a significantly increased density of immunoreactive astrocytes in the cerebral cortex after orchidectomy (Fig. 5). Androgens also profoundly affect astrogliosis after neural injury. Barreto et al.^145^ used an orchidectomized rat model to investigate and compare the effects of testosterone, estradiol, and non-aromatizable DHT treatment on reactive astroglia and microglial staining after a stab wound injury to the brain.

Early and delayed subcutaneous treatment with either testosterone or estradiol significantly reduced immunoreactive astrocyte and microglial staining within the hippocampus. Intriguingly, DHT had no effect on astrocyte staining but significantly reduced the volume fraction of immunoreactive microglial staining after early subcutaneous treatment. This suggests that early and late androgenic regulation of astroglia and microglia reactivity to neural injury is, at least in part, mediated through aromatization to estradiol whereas the early effects of androgens on microglia reactivity may also involve AR-specific mechanisms.

## Conclusions and Future Directions

Androgen-regulated neural plasticity may play a role in both the normal development and function of the brain and in pathological states, leading to the development of sexually differentiated neurological disease states. Although progress in understanding the mechanisms involved in these effects is less well developed than is the case for estrogen-induced neuroplasticity, in part because of the range of bioactive metabolites involved in testosterone's actions, there are hints that the effects of androgens may involve unique elements that are distinct from the effects of androgens on non-neural target tissues.

Understanding these mechanisms may help to clarify the effects of androgens on neurological disorders that are expressed more commonly in males than females, potentially helping to provide improved therapeutic modalities for these disorders. In concluding this review, we will briefly consider two elements of the neurotrophic actions of androgens in which significant gaps in our understanding remain, hopefully to stimulate additional research in these remaining areas of uncertainty.

A major unresolved question is: how the effects of testosterone on hippocampal synaptogenesis *in vivo* are mediated. These effects are clearly not mediated simply via conversion to estradiol.^94,22^ As discussed earlier, although testosterone induction of hippocampal spine formation is mimicked by DHT, suggesting AR involvement, it is also reproduced by the much weaker androgen DHEA—at doses that do not induce a significant peripheral androgenic effect.^95^

Administration of flutamide, at a dose sufficient to block the trophic effects of DHT on the prostate, does not antagonize, but rather potentiates the effects of this androgen on hippocampal spine synapse density (Fig. 3). In male rats with the Tfm mutation, which impairs AR function, the effects of both DHT and hydroxyflutamide are indistinguishable from those in normal wild-type males.^96^ Taken together, these observations suggest that the neurotrophic effects of androgens on hippocampal spine synapse density may be mediated via mechanisms distinct from those mediated through the “classical” intranuclear AR—despite the extensive evidence indicating the involvement of AR-linked kinase networks in the actions of DHT on spine plasticity in hippocampal neurons in culture.^93,100,101^

Exactly how androgens exert their effects *in vivo* remains unclear. We have previously speculated that the effects of testosterone *in vivo* may involve contributions from other mechanisms, such as potentiation of GABA-ergic transmission via the actions of 3α-diol.^94^ Contributions from afferent input originating from other parts of the brain may also be involved.^125,126^ Membrane AR-mediated responses may be involved,^101^ since this response pathway appears to be preserved in Tfm rats.^99^

A second area of uncertainty concerns the question of how the effects of gonadal steroids on neural plasticity are integrated within the preexisting neural circuitry. For steroid induction of neurogenesis and new synaptic connections to be beneficial, it needs to be integrated with the existing neural circuitry. Otherwise, it may have detrimental consequences—disrupting rather than enhancing function.^146^ How are new neurons integrated within existing neural networks? What processes ensure that androgen-induced increases in synapse density enhance function, rather than simply increasing “noise” in preexisting circuits?

An obvious possibility is that interactions between new neurons and potential synaptic targets, as has so elegantly been described for the SNB in the spinal cord,^147,148^ may direct functional connectivity in androgen-sensitive circuitry in the brain. In addition to growth factor-dependent regulation, however, it seems likely that mechanisms exist to regulate connectivity at the local level. What processes determine how newly formed synapses connect? Or are the mechanisms similar to those operating normally in development, with initial synaptic over-production being subsequently refined and focused by selective pruning of under-utilized connections?^149^

These questions cannot be answered definitively at the present time. One possibility that may merit further investigation, however, is that androgens may regulate cell–cell communication via the Wnt-β-catenin-cadherin signaling pathways. Wnts are critically involved in the organization of different cell types during development, regulating interactions between neighboring cells. A key component of the Wnt signaling pathway, β-catenin, also functions as a component of the cadherin complex, which controls cell–cell adhesion and influences cell migration.

Cell adhesion molecules, including the cadherins and protocadherins,^150^ are also of critical importance during embryonic development for morphogenesis of the central nervous system. This pathway is of particular interest, in the context of androgen action on the brain, for two reasons. First, the Wnt-β-catenin pathway is androgen-sensitive in the male reproductive tract,^151^ as well as in prostate cancer in which androgen regulation of the Wnt signaling contributes to the progress of the disease.^152^

Second, several recent studies have demonstrated that developmental neurological disorders such as autism spectrum disorder and schizophrenia, which occur more often in males than in females, are frequently associated with mutations in the cadherin signaling pathway.^153–155^ This raises the possibility that the relatively greater sensitivity of the male to these disorders might involve a genetic-hormonal interaction, with the effects of the mutations being enhanced by testosterone-induced responses.

Consistent with this hypothesis, Monks et al. have reported in rats that testosterone treatment increases expression of N-cadherin in both spinal motoneurons^156^ and the hippocampus.^157^ In the hippocampus, this effect was also induced by estradiol, but not by DHT,^157^ suggesting possible estrogen receptor involvement. Further studies of androgen regulation of the Wnt-β-catenin-cadherin signaling pathway may shed additional light on the mechanisms involved in androgen-induced neuroplasticity.

In summary, extensive evidence supports a role for androgens, locally synthesized in the brain as well as derived from adrenal and gonadal androgens delivered via the circulation, in the regulation of cellular plasticity in the central nervous system. This plasticity involves both androgen-induced enhancement of synapse formation and increases in the formation and survival of new neurons.

These effects may play a role in the normal development and function of the brain as well as in pathological states leading to the development of sexually differentiated neurological disorders. The underlying cellular mechanisms responsible for these processes, however, still remain incompletely defined. A better understanding of these mechanisms may allow the development of improved therapeutic interventions to correct the problems associated with androgen-sensitive neurological disease.

## Abbreviations Used

AD: Alzheimer's disease
AR: androgen receptors
BDNF: brain-derived neurotrophic factor
CREB: cAMP-response element binding protein
DG: dentate gyrus
DHEA: dehydroepiandrosterone
DHT: dihydrotestosterone
ERK: extracellular regulated protein kinase
fEPSP: field excitatory postsynaptic potentials
GDX: gonadectomized
GFAP: glial fibrillary acidic protein
GPER1: G-protein coupled membrane estrogen receptor 1
LTD: long-term depression
LTP: long-term potentiation
MAPK: mitogen-activated protein kinase
NMDA: N-Methyl-D-aspartate
PSD-95: post-synaptic density protein 95
SNB: spinal nucleus of the bulbocavernosus
Tfm: testicular feminization mutant
